# Pediatric anaphylaxis in the emergency department: Performances of dynamic tryptase measurement

**DOI:** 10.1016/j.jacig.2025.100524

**Published:** 2025-06-24

**Authors:** Constance Gonzalez, Meng Sun, Aurélie Morand, Philippe Minodier, Marc Leone, Jean-Christophe Dubus, Joana Vitte, Moïse Michel

**Affiliations:** a Aix-Marseille Université, Assistance publique–Hôpitaux de Marseille (AP-HM), Hôpital Timone, Service de Pneumologie et allergologie pédiatrique, Marseille, France; b Aix-Marseille Université, MEPHI, Institut Hospitalo-Universitaire Méditerranée Infection, Marseille, France; c Aix-Marseille Université, AP-HM, Hôpital Timone, Service d’accueil des Urgences pédiatriques, Marseille, France; d Aix-Marseille Université, AP-HM, Hôpital Nord, Service d’accueil des Urgences pédiatriques, Marseille, France; e Aix-Marseille Université, AP-HM, Hôpital Nord, Service d’Anesthésie et de Réanimation, Marseille, France; f Desbrest Institute of Epidemiology and Public Health, University of Montpellier, INSERM, Montpellier, France; g Immunology Laboratory, University Hospital of Reims, Reims, France; h University of Reims Champagne-Ardenne, INSERM UMR1250 P3CELL, Reims, France; i Department of Immunology, Nîmes University Hospital, Nîmes, France

**Keywords:** Acute tryptase, baseline tryptase, epinephrine, pediatric anaphylaxis

## Abstract

**Background:**

Dynamic measurement of serum acute (sAT) and baseline (sBT) tryptase confirms mast cell degranulation during systemic hypersensitivity reactions, provided timing and interpretation are appropriate. The current consensus formula requires sAT greater than a personalized cutoff value [sAT > (1.2 × sBT) + 2]. Only a few studies have investigated its diagnostic performance in children.

**Objective:**

We assessed the diagnostic accuracy of the consensus formula and alternative algorithms for interpreting tryptase levels in pediatric patients with suspected anaphylaxis.

**Methods:**

Medical records of suspected anaphylaxis referred to the pediatric emergency department of the University Hospitals of Marseille (France) from 2011 to 2020 were retrospectively reviewed. Clinical and laboratory data, including total tryptase and allergy evaluations, were collected. Anaphylaxis was defined as a sudden-onset, perceived life-threatening systemic reaction at the time of physician assessment. Inclusion criteria were suspected anaphylaxis and at least one tryptase determination. The diagnostic performance of the consensus formula was compared to 5 alternative tryptase interpretation algorithms.

**Results:**

Among 315 patients (median age, 7.8 years; 317 emergency department visits), 175 (55%) were categorized as cases. Food-induced anaphylaxis was diagnosed in 82%, and 92 (52.6%) had two or more tryptase determinations. The highest diagnostic performance was achieved by the sAT/sBT ratio. A cutoff for sAT/sBT ratio of >1.74 yielded 66.7% sensitivity, 90.0% specificity, 96.8% positive predictive value, and 24.3% negative predictive value. The retrospective study design was a major limitation.

**Conclusion:**

Compared to the current consensus formula, a sAT/sBT ratio above 1.74 may enhance the diagnostic performance of dynamic tryptase measurement in children with suspected anaphylaxis.

Anaphylaxis is an immediate and potentially life-threatening systemic reaction, most often due to mast cell (MC) degranulation, that represents a clinical and diagnostic challenge.^1^^,^^2^ International guidelines endorse the use of serum tryptase determination to confirm MC degranulation and thus to support the diagnosis of anaphylaxis.^2^, ^3^, ^4^, ^5^ However, most studies supporting this recommendation have been conducted in adults.^2^^,^^3^ The literature suggests that the diagnosis of pediatric anaphylaxis, which is more challenging than in adults, might be confirmed by tryptase determination.^6^^,^^7^

A 2012 international consensus proposal enshrined the adequate sampling times of serum acute tryptase (sAT) and serum baseline tryptase (sBT).^8^ It established the current consensus formula (CCF) that sAT values exceeding a personalized cutoff calculated as [(1.2 × sBT) + 2] confirm systemic MC activation (MCA) and degranulation.^8^ These findings were further confirmed in 2019.^9^

Data on tryptase performance for the diagnosis of pediatric anaphylaxis are scarce.^10^, ^11^, ^12^ A previous study including 41 children with sAT and sBT admitted to the emergency department (ED) found a 60.3% diagnostic sensitivity for the CCF, and up to 85.7% in severe reactions.^7^ In food-induced anaphylaxis in 49 children, serum tryptase determinations displayed 65.3% sensitivity.^13^

These studies applied the CCF to small pediatric cohorts and found moderate performance. We took advantage of a 10-year database on tryptase sampling at the pediatric ED of the University Hospitals of Marseille, France, to describe a large pediatric cohort of anaphylaxis and the performance of tryptase determination in this context.

## Methods

### Study population

Patients were retrospectively included in our cohort only if at least one tryptase determination was performed during the ED visit and was available from the laboratory database. Demographic, clinical, and laboratory data were collected for pediatric patients (18 years or younger) admitted with a suspicion of anaphylaxis from January 1, 2011, to December 31, 2020, at the ED of the University Hospitals of Marseille, France. For each patient, we collected data on age, sex, atopic history (asthma, allergic rhinitis, atopic dermatitis, food, drug, latex allergy), and presence of MC-related diseases (clonal MC diseases, MCA syndrome).^14^ At ED admission, the definition of anaphylaxis was a perceived life-threatening systemic reaction of sudden onset at the time of clinical evaluation by the physician in charge of the child’s care.^15^ Clinical manifestations were classified as mucocutaneous (rash or skin flushing, angioedema), respiratory (sneezing, bronchospasm, laryngeal edema or stridor), gastrointestinal (abdominal pain, tingling mouth, nausea and vomiting) and cardiovascular (hypotension, tachycardia, bradycardia) symptoms. The severity was graded using the 4-class scale adapted from Ring and Messmer.^16^ Grade 1 reactions were defined by the presence of mucocutaneous symptoms only. Grade 2 reactions were characterized by the presence of any of the previous symptoms as well as moderate respiratory or digestive symptoms. Cases were categorized as grade 3 if any of the following symptoms were present: cyanosis, hypoxia (saturation < 92%), hypotension, dysrhythmia, confusion, or loss of consciousness. In case of cardiac arrest, the reaction was classified as grade 4.

Patients received a state-of-the-art allergic assessment relying on clinical information and a set of complementary procedures, according to international and national guidelines. To reduce potential bias, the medical record of each patient was reviewed by two investigators of the study and, if necessary, by the physician who treated the child. The diagnosis of anaphylaxis was confirmed if the reaction involved at least one extracutaneous systemic manifestation and proof of systemic MCA (ie, transient serum tryptase elevation) or if proof of allergic sensitization to the culprit allergen had been obtained.^2^^,^^5^ Thus, patients meeting the diagnostic criteria for anaphylaxis were categorized as the anaphylaxis group (cases). Patients who did not meet the criteria were categorized as the control group.

### Ethics statement

Clinical and laboratory data were collected retrospectively using the institutional electronic medical records and deidentified before statistical analysis. The AP-HM institutional review board approved the study as nonhuman subjects research (PADS 2021-59). Under French law, ethics committee approval and patient consent were waived for this type of noninterventional study, provided the patients had received information about the potential use of anonymized medical data for research purposes and they retained the right to oppose it (SFAR CERAR committee IRB 00010254-2018-004; CIL/APHM registry 2017-24).

### Tryptase determination

Serum tryptase was measured using a fluorescent enzyme immunoassay ImmunoCAP platform (Phadia Thermo Fisher Scientific, Uppsala, Sweden) according to the manufacturer’s recommendations. The lower and upper limits of quantification were 1 and 200 μg/L. In line with international recommendations, we considered an adequate sampling time to be sAT sampled between 30 minutes and 2 hours after symptom onset and sBT sampled at least 24 hours after symptom resolution.^17^

### Tryptase interpretations and statistical analysis

For tryptase interpretation in patients with adequate sAT and sBT sampling times, we compared the manufacturer’s upper reference limit (11.0 μg/L during the study period), the CCF, and 2 other formulae (sAT > 1.35 × sBT; sAT > sBT + 3) according to previous data.^18^^,^^19^ We also evaluated the ratio (sAT/sBT) and the change (sAT − sBT) in tryptase (hereafter rT and dT, respectively) using receiver operator curve analysis, with the area under the curve to quantify the overall ability of the formulae to discriminate between anaphylaxis and control groups. Then the optimal thresholds maximizing both sensitivity and specificity were identified.

Statistical analysis was performed by GraphPad Prism v9.2.0 software (GraphPad Software, La Jolla, Calif). Results are expressed as medians with 5-95 percentiles. Mann-Whitney tests were performed to compare tryptase values and all numeric data between the two groups because of its suitability for nonparametric data, which does not assume a normal distribution. Significance was set at *P* < .05.

## Results

### Description of study cohort

The study cohort comprised 315 patients referred to the pediatric ED with a suspicion of anaphylaxis, with two children experiencing two distinct episodes, resulting in 317 events (Table I). The median (range) age was 7.8 (0.2-15.9) years with a male/female sex ratio of 1.3. Sixty-six patients (21.0%) had a history of asthma according to their general practitioner. Epinephrine was administered to 96 children (30.3%). Retrospective analysis, as described above, confirmed the diagnosis of anaphylaxis in 175 patients (55.2%; anaphylaxis group). The remaining 142 patients formed the control group. Skin symptoms affected 226 patients (71.3%), while grade 2 reactions represented 111 patients (63.4%).

**Table I:** Demographic and clinical characteristics of study cohort

Characteristic	Value
**Demographic information**	
Sample size	315
Age	
0-3 years	101 (32.1)
3-6 years	41 (13.0)
6-9 years	36 (11.4)
9-12 years	48 (15.2)
12-15 years	46 (14.6)
16-18 years	43 (13.7)
No. of episodes	317
No. with tryptase sampling	486
Mean no. of tryptase samples per episode	1.5
Age (years), median (5-95 percentile)	7.8 (0.2-15.9)
Sex ratio (M/F)	1.3 (177/140)
**Atopic, allergic, and MC-related conditions**	
Asthma	66 (21.0)
Atopic dermatitis	43 (13.7)
Allergic rhinoconjunctivitis	20 (6.4)
Known allergy∗	22 (7.0)
MC-related diseases	5 (1.6)
Symptoms per episode	
Mucocutaneous system	226 (71.3)
Respiratory system	125 (39.4)
Gastrointestinal system	105 (33.1)
Cardiovascular system	39 (12.3)
Anaphylaxis group†	175 (55.2)
Grade 1	20 (11.5)
Grade 2	111 (63.4)
Grade 3	31 (17.7)
Grade 4	0
Unknown	13 (7.4)
Control group	142 (44.8)

Data are presented as nos. (%) unless otherwise indicated.

∗ Refers to presence of physician-diagnosed food-, inhalant-, drug-, and/or Hymenoptera venom–induced allergy in patient’s known history.

† Anaphylaxis reactions are classified according to Ring and Messmer.^16^

### Comparison of anaphylaxis and control groups

We compared the patients in the anaphylaxis group (n = 175, 55.2%) with those in the control group (n = 142, 44.8%). Cutaneous (86.9% vs 52.1%, *P* < 10^−3^), lung (53.7% vs 21.8%, *P* < 10^−3^), gastrointestinal (46.3% vs 16.9%, *P* < .001), and cardiovascular (18.3% vs 4.9%, *P* < .001) symptoms were reported more frequently in the anaphylaxis group than in controls (Table II). Epinephrine was significantly more often administered in the anaphylaxis group (*P* < .001).

**Table II:** Comparison of clinical and laboratory data of episodes between anaphylaxis and control groups

Characteristic	Anaphylaxis	Control	*P* value	All
Sample size	175 (55.2)	142 (44.8)	—	317
Age, median (5-95 percentile)	6.3 (0.1-15.7)	9.1 (0.5-16.7)	.003	—
Sex ratio (M/F)	1.5 (105/70)	1.0 (72/70)	.11	—
**Symptoms**				
Mucocutaneous system	152 (86.9)	74 (52.1)	<.001	226 (71.3)
Respiratory system	94 (53.7)	31 (21.8)	<.001	125 (39.4)
Gastrointestinal system	81 (46.3)	24 (16.9)	<.001	105 (33.1)
Cardiovascular system	32 (18.3)	7 (4.9)	<.001	39 (12.3)
Epinephrine receipt	82 (46.9)	14 (9.9)	<.001	96 (30.3)
**Tryptase**				
Only 1 tryptase sample	83 (47.4)	110 (77.5)	<.001	193 (60.9)
At least 2 tryptase samples	92 (52.6)	32 (22.5)	<.001	124 (39.1)
Adequate sampling time∗	45 (48.4)	9 (28.1)	.04	54 (43.5)
Premature sAT (<30 minutes)	0	0	—	0
Delayed sAT (>2 hours)	24 (26.9)	8 (25.0)	.99	32 (25.8)
Premature sBT (<24 hours)	23 (24.7)	15 (46.9)	.02	38 (30.7)
Acute tryptase (sAT) (count)	174 (99.4)	135 (95.1)	.01	309 (97.5)
Acute tryptase (μg/L), median (5-95 percentile)	7.4 (2.7-25.4)	4.6 (1.6-11.1)	<.001	6.0 (2.0-21.5)
sBT count	58 (33.1)	17 (12.0)	<.001	75 (23.7)
All (μg/L), median (5-95 percentile)	3.9 (1.3-13.8)	4.3 (1.4-8.6)	.65	4.2 (1.4-11.5)
Samples with sBT > 8 μg/L	7 (12.1)	1 (5.9)	.47	8 (2.5)
sBT > 8 μg/L, median (5-95 percentile)	11.5 (9.6-18.0)	—	—	11.5 (9.6-18.0)

Data are presented as nos. (%) unless otherwise indicated.

∗ Defined as sAT sampled between 30 minutes and 2 hours after symptom onset and sBT sampled at least 24 hours after symptom resolution.

In the anaphylaxis group, at least two serum tryptase determinations were performed more often (52.6% vs 22.5%, *P* < .001), whereas a single serum tryptase determination was more frequent in the control group (77.5% vs 47.4 %, *P* < .001). Adequate sampling time as per international guidelines was more frequent in the anaphylaxis group than in the control group (48.4% vs 28.1%, *P* = .04). sAT was adequately measured in 99.4% of anaphylaxis patients and in 95.1% of controls (*P* = .01), and a higher serum concentration was reported in the anaphylaxis group (7.37 vs 4.57 μg/L, *P* < .001). Similarly, sBT was adequately sampled in 33.1% of anaphylaxis patients compared to 12.0% of controls (*P* < .001). However, sBT concentrations were similar (3.94 vs 4.26 μg/L, *P* = .65).

### Triggers of anaphylaxis and differential diagnosis

Most anaphylaxis triggers in this cohort, or 144 (82%) of 175, were foods: legumes, seeds, and nuts (36.0%, n = 58), cow’s-milk proteins (28.0%, n = 45), and miscellaneous foods (25.5%, n = 41). Drugs and Hymenoptera stings accounted for 17 events (10.6%), grouped together as nonfood allergens. In 14 cases, the trigger could not be identified.

The nature of the anaphylaxis trigger was associated with variations in the severity of reactions (*P* = .004). Indeed, we found grade 2 (79.3%) and grade 3 (15.5%) reactions with legumes, seeds, and nuts, while cow’s-milk proteins were associated with grade 2 (60.0%) and grade 1 (17.8%) reactions (Table III). sAT and sBT did not differ as a function of the trigger in the study cohort (*P* = .43 for both).

**Table III:** Comparison of clinical and tryptase determinations according to anaphylaxis triggers

Trigger	Legumes, seeds, nuts	Cow’s-milk proteins	Other foods	Nonfood∗	*P* value†
Sample size	58	45	41	17	
Anaphylaxis					.004
Grade 1	2 (3.5)	8 (17.8)	4 (9.8)	5 (29.4)	
Grade 2	46 (79.3)	27 (60.0)	22 (53.7)	6 (35.3)	
Grade 3	9 (15.5)	4 (8.9)	10 (24.4)	5 (29.4)	
Grade 4	0	0	0	0	
Unknown	1 (1.7)	6 (13.3)	5 (12.2)	1 (5.9)	
Acute tryptase (μg/L), median (5-95 percentile)	8.4 (4.7-15.2)	9.4 (3.1-25.3)	7.0 (2.4-21.5)	6.6 (1.8-49.4)	.4
Baseline tryptase, (μg/L), median (5-95 percentile)	4.0 (1.4–11.4)	4.5 (2.2-14.3)	3.1 (1.7-10.3)	4.3 (2.5-7.0)	.4

∗ Anaphylaxis triggered by drugs (n = 13) and Hymenoptera venom (n = 4).

† One-way ANOVA performed between subgroups of patients attending ED.

In the control group, several differential diagnoses were identified. The most common was an infectious disease (33.8%, n = 48), which could present with gastrointestinal and/or cutaneous symptoms, or even with cardiovascular signs in cases of septic shock. Additionally, severe acute asthma exacerbations (30.3%, n = 43), acute urticaria episodes (12.0%, n = 17), drug adverse effects mimicking anaphylaxis (11.3%, n = 16), and food poisoning (7.0%, n = 10) were also observed. Mastocytosis and MCA syndrome were also observed in the control group (5.6%, n = 8). In the control group, epinephrine was administered to 14 patients, all of whom were hospitalized for septic shock initially misdiagnosed as anaphylaxis, given the cardiovascular involvement and a credible history of exposure to a potential allergen.

### Performance of different decision thresholds for serum tryptase

To evaluate the performance of serum tryptase, we only considered episodes with an adequate sampling time and compared the patients with anaphylaxis to the control group. The CCF displayed 62.2% sensitivity and 80% specificity, with a Youden index of 0.42. Table IV details the results for each formula. Receiver operator curve analysis for rT and dT showed an area under the curve of 0.84 and 0.80, respectively, with 1.74 and 4.12 as optimal thresholds. At the optimal threshold, the sensitivity was 66.7% and 51.1% for rT and dT, with 90% specificity for both. rT was the best option to confirm anaphylaxis.

**Table IV:** Performance analysis of algorithm for tryptase dynamic evaluation with adequate sampling time

Characteristic	Anaphylaxis (no.)	Control (no.)	Sensitivity (%)	Specificity (%)	PPV (%)	NPV (%)	Youden index∗	Optimal threshold	AUC (95% CI)
sAT > (1.2 × sBT) + 2	45	9	62.2	80.0	93.3	32.0	0.42	—	—
sAT > 1.35 × sBT	45	9	82.2	60.0	90.2	42.9	0.42	—	—
sAT > sBT + 3	45	9	60	70.0	90.0	28.0	0.30	—	—
sAT > 11.0 μg/L	131	92	29.8	94.6	88.6	48.6	0.24	—	—
rT	45	9	66.7	90.0	96.8	37.5	0.57	>1.74	0.84 (0.72-0.96)
dT	45	9	51.1	90.0	95.8	29.0	0.41	>4.12	0.80 (0.64-0.96)

*AUC,* Area under the curve; *CI,* confidence interval; *NPV,* negative predictive value; *PPV,* positive predictive value; *rT,* ratio tryptase; *sAT,* acute tryptase; *sBT,* baseline tryptase.

∗ The Youden index is a statistical method used to determine the optimal threshold for diagnostic tests. It takes into account both sensitivity and specificity, aiming to identify the cutoff point that maximizes both parameters simultaneously. The closer the index is to 1, the better the overall test performance.

## Discussion

Pediatric anaphylaxis remains a challenging issue, underlined by the unmet need of specific guidelines. Here, using the description of a 10-year series of pediatric anaphylaxis in outpatients, we provide insight into pediatric anaphylaxis diagnosis in ED, with a focus on the use and interpretation of serum tryptase.

We described a cohort of 317 pediatric episodes (315 patients) of suspected anaphylaxis in a tertiary-care hospital, with 175 children (55.2%) confirmed as anaphylactic on retrospective review. Most of these patients experienced food-induced, milder reactions, in line with previous findings.^20^^,^^21^ Indeed, half of our cases were attributable to seeds, nuts, and cow’s-milk proteins, which are well-known allergens in children. Epinephrine, the first-line treatment for suspected anaphylaxis,^2^^,^^22^ was prescribed in only 96 (30.3%) of the suspected cases, and 14 were inadequately administered the drug to patients with septic shock with cardiovascular involvement. Several clinically salient features were noted in our 10-year pediatric ED cohort. Interestingly, no grade 4 pediatric anaphylaxis was observed. Drugs and insect stings were unfrequent triggers, demonstrated in less than 11% of the anaphylaxis events. A trigger could not be identified in only 8%, a relatively small proportion of anaphylaxis events. By collecting data, we found that 8 patients had an elevated sBT (above 8 μg/L), which could suggest the presence of an underlying MC-related disease, one not necessarily known at the time of the episode. Our results were in line with some,^23^^,^^24^ but not all,^25^ available data from various surveys.^26^

Our main objective was to evaluate the diagnostic performance of serum tryptase determination. In suspected anaphylaxis, two tryptase samples are required to confirm the diagnosis of anaphylaxis: sAT and sBT.^2^^,^^9^ These rules were barely followed in our center because a single sample was collected in roughly half of our patients. Actually, in a survey, Grossman et al^25^ showed that most physicians discharged patients after 4 or 8 hours of monitoring, making it impossible to complete sequential tryptase sampling with adequate timing. A previous local study showed that pediatricians intuitively adapted the treatment to the clinical severity of anaphylaxis, with a 4-fold increase in epinephrine administration in grade 3 compared to grade 2 anaphylaxis.^26^ When at least two samples of tryptase were collected, the sampling timing was adequate in only half of the cases. Taken together, our study underlines the need for improved adherence to guidelines by better education of ED medical staff. A before-and-after comparative study suggested the relevance of an updated protocol to improve the anaphylaxis management in a pediatric ED.^27^

We evaluated 3 different formulae for the interpretation of dynamic serum tryptase. In our hands, the CCF was not the best option. Indeed, we found a better sensitivity (82.2%) with the [sAT > 1.35 × sBT] formula and a better specificity (94.6%) with the [sAT > 11.0 μg/L] formula. Overall, the best formula was the rT—that is, the ratio of sAT over sBT, with 66.7% sensitivity, 90.0% specificity, and 0.57 Youden index. All these formulae exhibited high positive predictive values, but none of them had a high negative predictive value, which is an important limitation in clinical practice. However, because of our strict analysis conditions (adherence to adequate tryptase sampling times), we only analyzed a small number of case samples. All these results must be confirmed in larger cohorts with a prospective design to minimize potential biases. A previous study, which analyzed sAT and sBT to discriminate IgE-dependent from non–IgE-dependent hypersensitivity reactions in both children and adults using rT,^28^ found that the ratio was higher in IgE-mediated reactions. Moreover, rT and dT were previously evaluated on 12 shrimp-related pediatric anaphylaxis episodes, and at optimal thresholds, the authors found 92% and 83% sensitivity, and 96% and 93% specificity, respectively.^29^ As a whole, rT displayed convincing results and appears to be the best option for pediatric anaphylaxis, notably because of its high specificity, which helps avoid falsely positive results. Having a more accurate formula for documenting systemic MCA in children may improve overall patient management. Clinical practice implementation of a formula with validated performance ensures better characterization of anaphylactic reactions, patient referral for allergy assessment, and improved prevention of future episodes. In a multicenter cohort of suspected pediatric perioperative hypersensitivity, we did not find that the CCF was the best option to document MCA. Instead, sAT values exceeding a cutoff of sBT + 0.71 displayed 53.2% sensitivity and 96.9% specificity.^30^

One probable explanation for the performance variations between adult and pediatric populations lies in the fact that baseline tryptase secretion of MC significantly evolves throughout childhood. Indeed, several studies aiming to establish age-specific reference values of sBT have shown a marked decrease between ages 9 and 19, followed by a gradual increase into adulthood.^31^^,^^32^

Overall, data from our study suggest that the CCF may not be the most suitable formula for documenting MCA. Here, we highlight a plausible option for pediatric populations referred to the ED. The performance might vary according to factors such as age (pediatric or adult), sex,^33^ clinical context, type of suspected allergen, or presence of comorbidities, most of which are much less prevalent in children than in adults, including chronic renal disease,^9^ myeloid neoplasm,^9^ MC-related diseases,^34^ and obesity.^35^

The major strength of our study is the real-world data described in a specific population of pediatric patients. To date, our study represents the largest cohort analysis on sequential tryptase performance in pediatric anaphylaxis outside the perioperative setting.^6^^,^^36^ The major weaknesses of our study was its retrospective, single-center design and the absence of grade 4 cases of anaphylaxis. The lack of demographic matching with a control group is a potential limitation of our study, but this can be explained by the nature of our study, which focuses on the analysis of real-life data.

Our study underlines the need for a better implementation of both sAT and sBT measurements at adequate sampling times in pediatric ED and contributes to the ongoing debate regarding the optimal interpretation of pediatric dynamic tryptase. Similar to adult sAT and sBT, pediatric laboratory documentation of MCA supports the demonstration of pediatric anaphylaxis, contributes to uncovering disease-modifying MC-related conditions, and ultimately improves the management of pediatric anaphylaxis.

In conclusion, pediatric anaphylaxis is an at-risk clinical condition that is frequently suspected in the ED. Improving the knowledge on the kinetics of serum tryptase concentrations for pediatric patients should result in improved management of these patients. We provided here some key points about this topic through our single-center experience. Further studies are required to confirm our observations about pediatric anaphylaxis in order to improve patient management.

## Disclosure statement

Disclosure of potential conflict of interest: J. Vitte reports receipt of speaker and consultancy fees from AstraZeneca, HpVac, Novartis, L’Oréal, Sanofi, and Thermo Fisher Scientific outside the submitted work. The rest of the authors declare that they have no relevant conflicts of interest.
